# Concussion in motorsport: incidence, awareness and future directions

**DOI:** 10.2217/cnc-2017-0004

**Published:** 2017-07-06

**Authors:** Naomi D Deakin, Peter J Hutchinson

**Affiliations:** 1 Department of Neurosurgery, Addenbrooke's Hospital, Cambridge, CB2 0QQ, UK; 2 Academic Division of Neurosurgery, Addenbrooke's Hospital, University of Cambridge, Box 167, Cambridge, CB2 0QQ, UK

**Keywords:** concussion, mild TBI, motorsport

Concussion in contact sport is a contentious issue and represents a growing proportion of injuries sustained by athletes worldwide. Motorsport is no exception, yet the published evidence on the incidence, severity and recovery from this condition remains scarce [1]. Motorsport is unique among extreme sports, since competitors are frequently subject to high velocity, high G forces with a large rotational component, even without impact [2,3]. The ongoing challenge for practitioners is threefold; making a diagnosis of concussion trackside, evaluating competitors in the medical center and approaching the return-to-race decision.

## Concussion in motorsport

When compared with other high-risk sports, published data illustrate that drivers have a greater risk of concussion; calculated as 181/100,000 participants with adjustment for national sports participation figures [4]. However, the incidence of motorsport concussion in the literature varies from 6.3 to 25%. These studies are difficult to compare, ranging from local to national events, lasting from 1 day to over a decade and including tens, or tens of thousands of individuals [5]. In addition, competition-specific figures are scarce but are most well defined for motocross, where concussion is highlighted as one of the most common injuries sustained, with presentations ranging from 9.5 to 29%. Far less data are published for professional motorsport and estimates range from 1.6 to 17.6%, with study duration of single events to multiple racing seasons [2,6–9].

Recent unpublished data from the British Touring Car Championship indicate that the incidence of concussion may be increasing. A total of 72 drivers underwent baseline neuropsychological testing during the 2015/2016 season, with 50 formal diagnoses of concussion made in the preceding 35-year period. The distribution of these diagnoses was striking; 2 drivers during the 1980s and 7 during the 1990s, compared with 21 drivers from 2000 to 2009 and 20 drivers in the 6 years from 2010. These data are skewed by current participation, but highlight the need to accurately ascertain the true prevalence of concussion in motorsport.

## The assessment of concussion in motorsport

As an aid to clinical diagnosis, various sideline screening tests are utilized in motorsport. The most popular is the Sport Concussion Assessment Tool version 3 (SCAT3); mandated by the Fédération Internationale de Motocyclisme (FIM) [10]. There remains some use of the Sport Concussion Assessment Tool version 2 (SCAT2) and minimal uptake of the King–Devick test.

When evaluating competitors in the medical center or beyond, clinical decision making for fitness to participate is supported by the Immediate Post-Concussion Assessment and Cognitive Test (ImPACT), a computerized neurocognitive test created specifically for use in athletes. A 45-min battery of neuropsychological tests is utilized to evaluate memory, visuomotor speed and reaction time, using a desktop or Internet-based program. ImPACT has been employed without formal validation in motorsport since the early 2000s and was first formally mandated by NASCAR in 2014. The use of ImPACT is now mandated by the Federation Internationale de l'Automobile (FIA) [11], and is advised by the FIM [10], AMA ProRacing [12] and Indy Car [Dr Stephen Olvey, Doctor for Indycar, Pers. Comm.].

## Competitor & clinician awareness

Few studies address the self-reporting of concussion in motorsport [13] and many believe these figures are underdocumented, especially in professional competition where the inability to participate can result in financial penalty. Despite this, a quarter of professional drivers in a single American study confirmed a diagnosis of concussion at some point in their career; this being the second most common injury-related fear among the participants [14]. Furthermore, a recent survey of motorsport competitors formally highlighted poor awareness of concussion among participants [15]. A total of 163 participants replied to the online survey from 31 countries (capturing medical professionals and competitors). Out of the 32 competitors surveyed, 45% had suffered a concussive episode but only 50% of these were discussed with a doctor. Less than a third of competitors (28%) withdrew voluntarily from their event and 70% did not feel completely normal when they attempted to return to race. The latter result is of great concern for motorsport. In contrast to many other sports, competitors returning to race remain in control of a high-speed vehicle which may pose a threat to the individual, other competitors, staff and spectators; the latter numbering hundreds of thousands at more popular international events, such as the FIA Formula 1 British Grand Prix [16]. Encouragingly, three quarters of competitors expressed an interest in learning more about concussion; a want that must be capitalized upon in the short term.

## Return to race guidance

In the study described above over 100 medical professionals provided online responses (n = 130; 80% of the total cohort). Ninety percent of the medical staff had clinical experience of a concussed competitor and 50% had seen more than five individuals with the diagnosis. Almost the entire cohort wished for more formal guidance regarding the management of concussion and barely a third had access to guidelines for hospitalization (none of which were motorsport specific).

In sports generally, return to participation decisions after concussion are currently governed in the broadest sense by the Zurich 2012 update of the Concussion in Sport Group guidelines [17]. In motorsport, these guidelines are followed explicitly by the FIM Medical Code [10]. The UK Motor Sports Association has its own concussion guidelines, which mandate an exclusion period of 14–21 days for all driving activity, after which the driver must pass a full clinical assessment before their license is returned [18]. To date, there is no formal published guidance from the FIA, perhaps contributing to the international desire for formal protocols highlighted by the above study.

## Future perspective

In contrast to the vast financial investment of engineering frontiers in motorsport, the allocation of resources to medical research in the sport remains minimal. There are, however a small number of ongoing projects, focusing on the trackside diagnosis of concussion and the ongoing management of concussed competitors.

### Trackside diagnosis

Concussion is defined clinically according to a constellation of symptoms and motorsport-specific research illustrates that this generic symptomatology may persist throughout the racing season [19]. Novel adjuncts to clinical assessment include the introduction of eye tracking and vestibular assessment tools, both of which are currently being investigated in IndyCar and the British Touring Car Championship.

### Ongoing management of concussed competitors

There exists a wide spectrum of individual heterogeneity in the severity of concussion and its rate of recovery, with acknowledged differences in females and adolescents, both of whom represent growing participant groups in motorsport. Frontiers in neuroimaging indicate that cerebral function may be based upon inter- rather than intraregional communication [20], which may be disturbed by the exertion of mechanical forces upon the head. Future advances in motorsport may therefore focus on the use of specialized neuroimaging, such as MRI. The latest scanners can achieve resolutions of up to 100 μm, with the resultant ability to visualize the cerebral anatomy and its connectome in exquisite detail. Specific applications may include the use of susceptibility- or diffusion-weighted imaging, in addition to multiparametric mapping and functional series. Furthermore, little is known about how currently used computerized neurocognitive tests, such as ImPACT, relate to structural or functional neurobiological changes of head injury and care must be taken in how these tools are used to monitor recovery and return-to-race decisions in the sporting world.

## Conclusion

Concussion is a hot topic for medical professionals and a real fear for competitors in motorsport, yet its incidence and prevalence remain poorly documented in the published literature. Commonly utilized computerized assessment tools have not been validated for use in motorsport and more specific instruments are required. Finally, there remains a user need for formal guidance regarding return-to-race decisions in motorsport. The 2016 update to the Concussion in Sport Group guidelines is awaited and the authors hope to see this material utilized to create new concussion protocols for all world-wide organizations, in addition to their incorporation into existing documents.

American data have demonstrated that rates of concussion may be reduced by collaboration with engineering colleagues, as exemplified by the sustained reduction of concussion seen in IndyCar after the modification of in-car padding [21]. Such progress should serve as an example of the possible successes that could be achieved with further research and investment in this growing field. The authors suggest that in-depth baseline and post-concussion studies in motorsport, such as neuroimaging, would provide a standard against which current neuropsychological assessments could be validated, both immediately post-injury and during the recovery period. In addition, the correlation of these data with real-time in-car accelerometer data may further assist in elucidating the exact mechanisms and thresholds for a concussive event, which could translate to improved care for the lay public involved in road traffic collisions worldwide.
